# Transcriptomic profiling reveals molecular regulation of seasonal reproduction in Tibetan highland fish, *Gymnocypris przewalskii*

**DOI:** 10.1186/s12864-018-5358-6

**Published:** 2019-01-03

**Authors:** Fei Tian, Sijia Liu, Jianquan Shi, Hongfang Qi, Kai Zhao, Baosheng Xie

**Affiliations:** 1 Key Laboratory of Adaptation and Evolution of Plateau Biota, Qinghai Province Key Laboratory of Animal Ecological Genomics, Northwest Institute of Plateau Biology, Chinese Academy of Sciences, Xining, Qinghai China; 2 University of Chinese Academy of Sciences, Beijing, 100049 China; 3 The Rescue and Rehabilitation Center of Naked Carps in Lake Qinghai, Xining, Qinghai China; 4 State Key Laboratory of Plateau Ecology and Agriculture, College of Ecol-Environmental Engineering, Qinghai University, Xining, Qinghai China

**Keywords:** Seasonality, Reproductive migration, Neuroendocrine, Day-length, RNA-seq, WGCNA, Tibetan highland fish

## Abstract

**Background:**

The Tibetan highland fish, *Gymnocypris przewalskii*, migrates from Lake Qinghai to its spawning grounds every summer. This seasonal reproduction is critically regulated by intrinsic and extrinsic signals. However, the molecular mechanisms that process environmental oscillations to initiate the seasonal mating are largely unknown.

**Results:**

A transcriptomic analysis was conducted on the brain and gonad of male and female *G. przewalskii* in reproductive and nonreproductive seasons. We obtained 2034, 760, 1158 and 17,856 differentially expressed genes between the reproductively active and dormant female brain, male brain, ovary and testis. Among these genes, *DIO2* was upregulated in the reproductively active brain and gonad of both males and females. Neuroactive ligand-receptor genes were activated in male and female brain. Functional enrichment analysis suggested that retinol metabolism was uniquely stimulated in reproductively active males. Genes involved in GnRH signaling and sex hormone synthesis exhibited higher expression levels in brain and gonad during the reproductive season. A co-expression network classified all the genes into 9 modules. The network pinpointed *CDC42* as the hub gene that connected the pathways in responsible for modulating reproduction in *G. przewalskii*. Meanwhile, the sex pheromone receptor gene *prostaglandin receptor* was identified to link to multiple endocrine receptors, such as *GnRHR2* in the network.

**Conclusions:**

The current study profiled transcriptomic variations between reproductively active and dormant fish, highlighting the potential regulatory mechanisms of seasonal reproduction in *G. przewalskii*. Our data suggested that the seasonal regulation of reproduction in *G. przewalskii* was controlled by the external stimulation of photoperiodic variations. The activated transcription of neuroendocrine and sex hormone synthesis genes contributed to seasonal reproduction regulation in *G. przewalskii*, which was presumably influenced by the increased day-length during the breeding season.

**Electronic supplementary material:**

The online version of this article (10.1186/s12864-018-5358-6) contains supplementary material, which is available to authorized users.

## Background

Seasonal reproduction is an evolutionary adaptive strategy for animals living in areas with evident seasonality [1–3]. To maximize the survival of offspring, animals in the mid and high latitudes reproduce at the most beneficial time of the year, which has moderate climate and sufficient food [2]. In vertebrates, the timing of mating behavior is tightly controlled by the coordination of both environmental cues and internal factors. Seasonal changes in temperature, food availability, and day-length are environmental factors required to initiate reproduction in many animals [4, 5]. Based on the relative day-length during the reproductive season, animals are classified into long-day (LD) and short-day (SD) breeders [2]. The light information received by the retina of mammals and extra retina tissues of lower vertebrates influences neuroendocrine production, transmitting environmental information to affect the physiological activities of animals, including reproduction [6]. For LD breeders, increased day length induces gonadotropin-releasing hormone (GnRH) production, which facilitates follicle-stimulating hormone (FSH) and luteinizing hormone (LH) synthesis as well as gonad development [7, 8]. The mechanisms controlling seasonal reproduction have been firmly established in mammals, which involve the stimulation of gonad development via the hypothalamic-pituitary-gonadal (HPG) axis depending on the seasonal fluctuation of melatonin production due to the day-length change [9].

Seasonal reproduction is widely observed in temperate fish species as well [10]. For example, medaka is characterized as a LD breeder that develops gonads from spring to summer, while salmonid fishes are SD breeders who migrate back to home rivers for spawning in autumn [11, 12]. Although seasonal reproduction is pervasive in fish, its molecular basis remains ambiguous. Saccus vasculosus (SV), a specific organ in fish brain that serves as a sensor responding to photoperiodicity, expresses all the components in the regulation of the photoperiodic signaling pathway [13, 14]. Meanwhile, a variety of neurotransmitters, such as melatonin, dopamine, acetylcholine, GABA, and Kiss1, as well as their receptors, have been reported to translate environmental stimuli into internal signals and synchronize reproduction in different fish species [10, 15–17]. Therefore, seasonal reproduction is likely to be controlled by multiple neuroendocrine molecules in fishes in response to photoperiodicity, with regulatory mechanisms distinct from those of mammals.

**Fig. 1:**
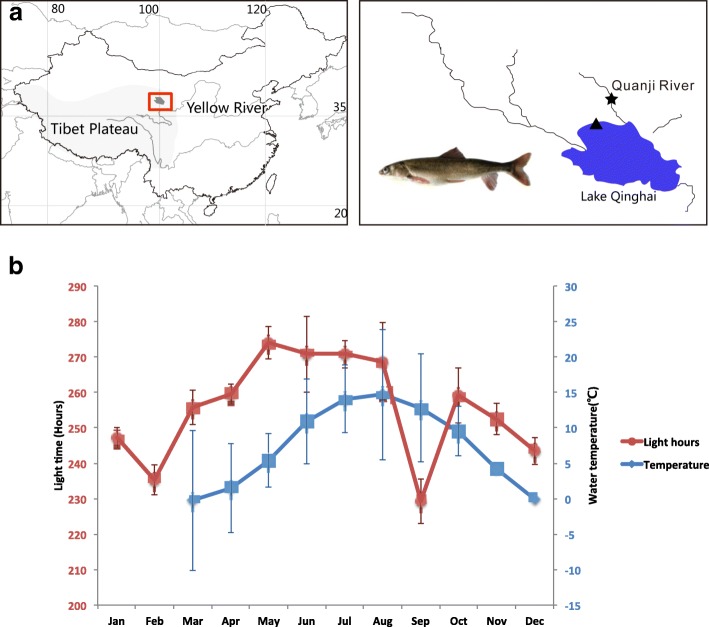
**a** Sampling sites. The sampling map was created using ArcGIS v10.1 (ESRI, CA, USA), and processed using Adobe Illustrator CS5 (Adobe System Inc., San Francisco, CA, USA). Data used in the map was downloaded from National Science & Technology Infrastructure of China (http://lake.geodata.cn) and http://www.statsilk.com/maps. Fish photo in (a) belongs to Dr. Kai Zhao. **b** Annual water temperature (y-axis on the right) and hours of light time (y-axis on left). The water temperature was measured about 0–0.7 m beneath the water surface of the Lake Qinghai. No data on water temperature was available in Jan and Feb due to the icing in the winter of the Lake Qinghai. The light hours reach the peak in May, and keep as the long day during from May to Aug. The decrease in light hours from Jun to Aug is primarily due to the rainy season in the summer. The environmental and ecological data collected from 1988 to 2004 [20]

*Gymnocypris przewalskii* (subfamily Schizothoracinae) is an endemic fish species living in Lake Qinghai in the Tibetan Plateau (TP), the largest saline and alkaline lake of China [18, 19]. Sexually mature *G. przewalskii* (over 3 years old) migrate to spawning rivers in late spring and summer (from May to August, the reproductive season, RS) and inhabit in the lake in early fall and winter (from September to April, the nonreproductive season, NRS). Since the water temperature fluctuates dramatically [20, 21], day-length is assumed to be the reliable environmental stimulus initiating courtship behavior in Tibetan highland fish (Fig. 1).

In the current study, we attempted to examine the role of the transcriptional regulation in controlling seasonal reproduction in *G. przewalskii.* To achieve this goal, brain and gonad samples from both male and female *G. przewalskii* were collected in the RS and NRS. Gene expression analysis revealed that the number of differentially expressed genes (DEGs) was 2034 in female brain, 760 in male brain, 1158 in ovary and 17,856 in testis genes between fishes in RS and NRS. DEGs were enriched in pathways including steroid hormone synthesis, neuroactive ligand-receptor interaction, and retinol metabolism. Gene co-expression network analysis revealed *CDC42* as a hub bridging genes in reproduction-related pathways. Taken together, our results showed that the timing of breeding in *G. przewalskii* was highly likely to be controlled by the increased day length in the summer, which probably induced the transcription of genes in neuroendocrine regulation of sex hormone synthesis.

## Results

### De novo assembly and annotation of *G. przewalskii* transcriptome

**Fig. 2:**
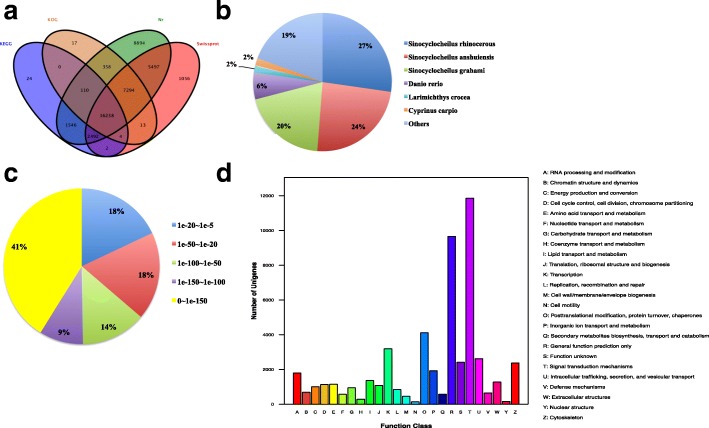
Annotation information of the *G. przewalskii* reference transcriptome. **a** Annotated unigenes by 4 public libraries. **b** Percentage of species similarity. **c** E-value distribution. **d** Functional classification of unigenes by the KOG database

To identify genes that may be involved in the reproductive migration of *G. przewalskii*, we performed transcriptomic sequencing of brain and gonad from 3 male and 3 female *G. przewalskii* collected in the RS and NRS. In total, 1,291,024,718 raw reads were generated from 24 libraries, which yielded 1,258,421,646 clean reads after the quality control procedure. The RNA-seq results were deposited in NCBI Sequence Read Archive (SRA) (SRP136464). The de novo assembled transcriptome included 122,750 unigenes with N50 of 1593 bp and an average length of 857 bp (Table 1). The reference transcriptome of *G. przewalskii* unigenes was annotated by 4 public databases, Nr, KOG/COG, Swiss-Prot, and KEGG. The results showed that 14.40% of unigenes were annotated by all databases, and 38.62% of unigenes were annotated by at least one database (Fig. 2a and Table 2). The Non redundant (NR) annotation demonstrated that 71% of unigenes were annotated by genes of 3 fish species (*S.rhinocerous*, *S. anshuiensis*, and *S. grahami*) in *Sinocyclocheilus*, the taxonomically closest fish species to Schizothoracinae (Fig. 2b). More than half of the annotated unigenes had E-values less than 1e-150, suggesting high sequence similarity between *G. przewalskii* and *Sinocyclocheilus* genomes (Fig. 2c). Functional annotation by KOG database indicated that 52,285 *G. przewalskii* unigenes were classified into 26 groups, with 11,862 unigenes involved in signal transduction mechanisms (Fig. 2d and Additional file 1: Table S2). KEGG annotation also showed that 3239 unigenes were mapped to 30 pathways related to environmental information processing, which were likely to regulate the timing of seasonal reproduction (Additional file 2: Table S3). The assembly and annotation of *G. przewalskii* reference transcriptome laid a solid basis for identifying genes involved in the control of seasonal reproduction.

**Table 1:** Summary of de novo assembly using Trinity

	Trinity
Number of raw reads	1,291,024,718
Number of clean reads	1,258,421,646
Number of unigenes	122,750
N50 of unigenes (bp)	1593
Average length of unigenes (bp)	875

**Table 2:** Annotation statistics

	Number of unigenes	Percentage (%)
Annotated in Nr	42,429	37.63%
Annotated in KOG	32,596	28.91%
Annotated in Swiss-Prot	24,034	21.32%
Annotated in KEGG	20,416	18.11%
Annotated in all databases	16,238	14.40%
Annotated in at least one database	43,545	38.62%

### Quantification of transcriptional abundance between RS and NRS samples

**Fig. 3:**
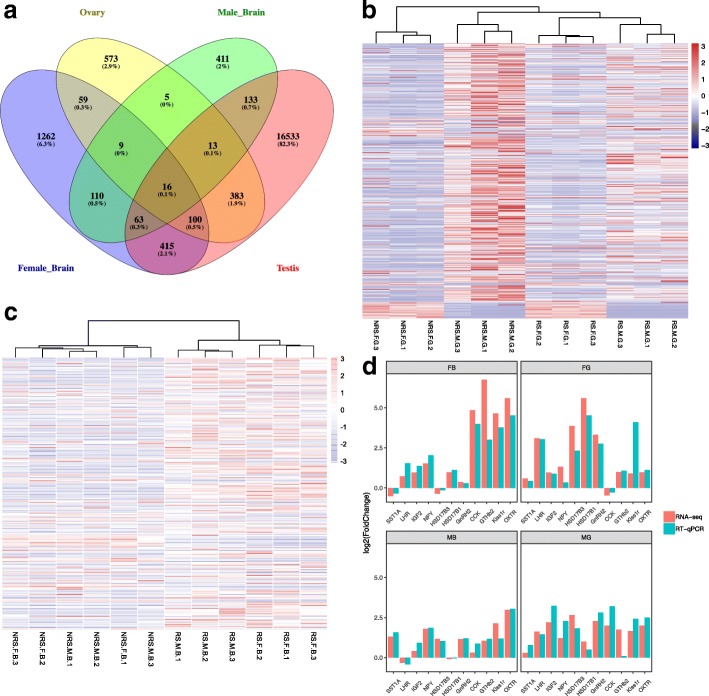
Analysis of DEGs between NRS and RS. **a** Number of DEGs shown by Venn diagram. (**b-c**) Cluster analysis and heatmap in gonad **b** and brain **c** of male and female samples from the RS and NRS. **d** RT-qPCR validation of DEGs obtained by RNA-seq. The y axis represents the log2FoldChange(RS/NRS). RS and NRS represent the average expression values of 3 biological replicates from each group

Using an adjusted *p*-value less than 0.05 and an absolute fold change greater than 2 as the threshold, 2034, 760, 1158 and 17,856 differentially expressed genes (DEGs) were discovered in female brain, male brain, ovary and testis of *G. przewalskii* between RS and NRS (Table 3). Interestingly, many more DEGs were found in testis than in other tissues, implying the significance of gene expression regulation in male gonad development. Surprisingly, we found a few DEGs that overlapped between male and female in both brain (7.63%, 198 DEGs) and gonad (2.77%, 512 DEGs), and 16 DEGs were shared by all 4 comparisons (Fig. 3a and Additional files 3, 4, 5 and 6: Tables S4-S7). Among these 16 common DEGs, 14 genes were upregulated in the reproductively active samples, including type II iodothyronine deiodinase (*DIO2*, Unigene0058594). For the 518 DEGs common to male and female gonads, a heatmap clearly displayed obvious differences among the 4 groups (Fig. 3b). In brain, the signatures of 198 shared DEGs clearly separated NRS and RS samples (Fig. 3c). Meanwhile, we found that these shared DEGs showed gender-dependent transcription patterns in RS but not in NRS, suggesting gender-specific regulation of reproduction in *G. przewalskii* (Fig. 3c). Moreover, male and female RS fish showed distinct expression patterns, suggesting that seasonal reproduction was probably controlled by different genes in male and female *G. przewalskii*. To validate the DEGs obtained from RNA-seq, the fold changes in 11 unigenes were measured using RT-qPCR, and the high correlation coefficient of 0.723 between the two methods indicated the accuracy and reliability of the RNA-seq results (Fig. 3c).

**Table 3:** Number of DEGs between the NRS and RS in female and male fish

Comparison	Total	Up	Down
MB	760	509	251
FB	2034	854	1180
MG	17,856	9974	7882
FG	1158	794	364

*MB* Male brain, *FB* Female brain, *MG* Male gonad, *FG* Female gonad, *Up* Upregulation in RS, *Down* Downregulation in RS

### Functional enrichment analyses of DEGs

**Fig. 4:**
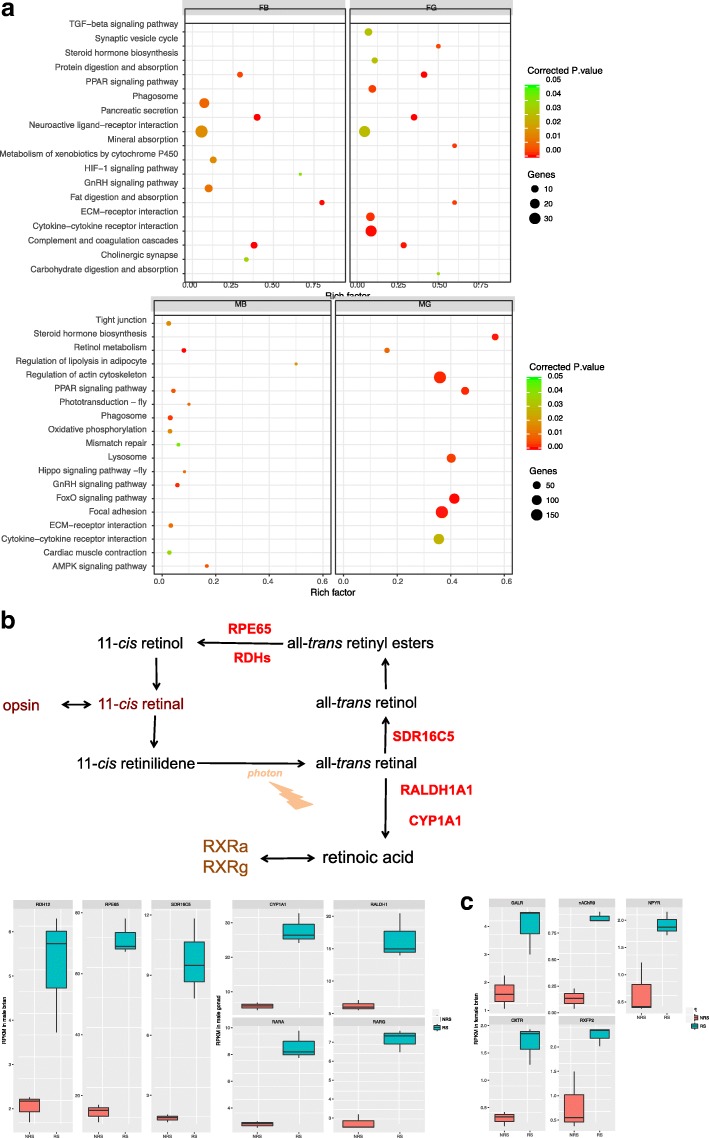
Functional enrichment of DEGs. **a** KEGG classifications of DEGs from brain and gonad. **b** Transcriptional comparison of genes in retinol metabolism in brain and testis between NRS and RS. **c** Expression levels of genes in neuroactive ligand-receptor interaction

To understand the functional consequences of expression variations, DEGs of male and female fish were mapped to KEGG pathways. The significantly enriched pathways were listed in Fig. 4a. Notably, GnRH signaling pathway and steroid hormone biosynthesis were identified as significant pathways in brain and gonad, respectively, indicating the orchestrated expression of genes in brain and gonad in controlling seasonal reproduction in *G. przewalskii*. In GnRH signaling pathway, the expression of *GnRH2* (Unigene0047142) and *LHβ* (Unigene0104459) was significantly promoted in male and female brain (Fig. 3b). In the steroid hormone biosynthesis pathway, *testosterone 17-β-dehydrogenase 3* (*HSD17B3*, Unigene0005059) and *estradiol 17-β-dehydrogenase* 1 (*HSD17B1*, Unigene0063161), key enzymes in the synthesis of testosterone and estradiol, showed enhanced expression levels in reproductively active fish gonads (Fig. 3b). Retinol metabolism was enriched in male brain and gonad, suggesting its essential role in regulating reproduction in males. Retinol metabolism contained 7 DEGs, including the activation of 3 genes in reproductively active male brain and 4 genes in RS testis (Fig. 4b). Additionally, neuroactive ligand-receptor genes exhibited gender- and tissue-specific activation patterns. For instance, *KISS1 receptor* gene (*Kiss1r*, Unigene0009160) was stimulated in RS male brain, and the expression of *LH receptor* (*LHR*, Unigene0086623) was increased in reproductively active gonads in both male and female individuals (Fig. 3b). In particular, we found that the mRNA levels of *neuropeptide Y receptor* (*NPYR*, Unigene0080609), *acetylcholine receptor 9* (*nAChR9*, Unigene0041188), *relaxin receptor 2* (*RXFP2*, Unigene0021440), *galanin receptor 2* (*GALR2*, Unigene0054699) and *oxytocin receptor* (*OXTR*, Unigene49178) were exclusively induced in RS female brain, suggesting their roles in the seasonal reproduction in female individuals (Fig. 4c). Not surprisingly, genes in metabolic and immune pathways were differentially expressed between fish in the RS and NRS. This result was highly likely to be related to the physiological and behavioral changes in *G. przewalskii* during its reproductive migration from the salt lake to the freshwater river.

### Identification of a seasonal-reproduction-related coexpression network

**Fig. 5:**
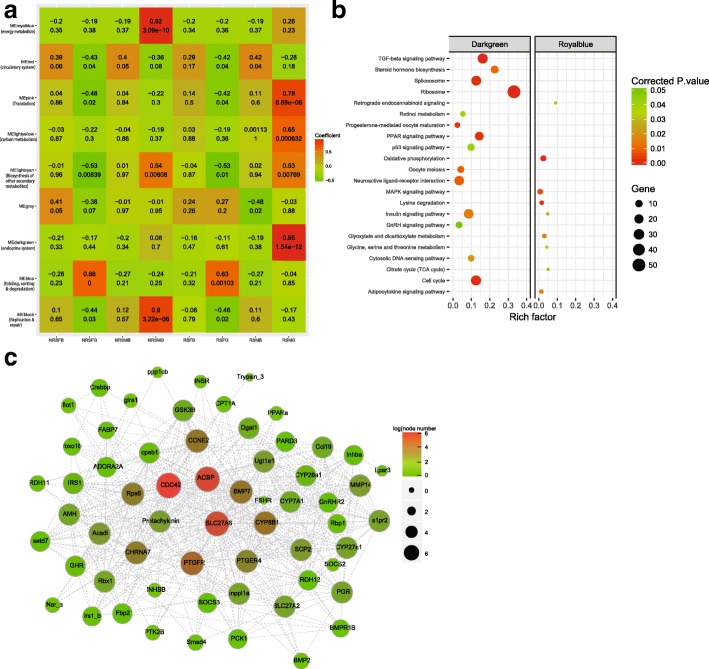
Construction of the coexpression network. **a** Correlation coefficients between each group and the gene expression in the module. The x-axis and y-axis represented the group and the module, respectively. The secondary KEGG categories that most significantly enriched pathways belonged to were labeled in the parentheses under module names. The grey module contained genes that were unable to classify into any other modules, therefore, the enrichment analysis was not conducted in genes of the grey module. The red and green colors indicated the highest positive and negative correlation between sample groups and modules. The correlation coefficient (1st line) and *p*-value (2nd line) were labeled. NRSFB: 3 samples of female brain in NRS; NRSFG: 3 samples of female gonad in NRS; NRSMB: 3 samples of male brain in NRS; NRSMG: 3 samples of male gonad in NRS; RSFB: 3 samples of female brain in RS; RSFG: 3 samples of female gonad in RS; RSMB: 3 samples of male brain in RS; RSMG: 3 samples of male gonad in RS. **b** KEGG functional enrichment of genes in the darkgreen and theh royalblue modules. **c** Network of genes in the darkgreen module with weight greater than 0.25. The node and edge represent the gene and weight between two genes. The red and green colors of the nodes indicated the high and low connectivity to other nodes. The larger node size also denoted the higher connectivity as well. The hub genes were labeled in red

With the goal of figuring out the networks involved in the mating regulation of *G. przewalskii*, weighted gene coexpression network analysis (WGCNA), a technique based on pairwise correlations between gene expression trends across all samples, was employed. Based on the expression values, we ultimately obtained 9 modules, and the genes within each module were highly interconnected according to their transcriptional levels (Fig. 5a). Genes in 9 modules showed distinct expression patterns across all the samples, among which pink and darkgreen as well as black and royalblue modules exhibited higher gene expression in RS and NRS male gonad (Additional file 7: Figure S1). According to the correlation coefficients between the module and the group, we found darkgreen and royalblue modules showed highest and significant correlation to RS and NRS male gonad (Fig. 5a and Additional file 8: Table S9). Therefore, we performed the KEGG enrichment analysis on genes in the two modules. In the royalblue module, 192 genes were enriched in pathways related to energy metabolism, presumably because of fasting during the spawning migration (Fig. 5b). In the darkgreen module, 2291 genes were classified into 14 significantly enriched pathways. We defined the darkgreen module as “endocrine system” since a large number of pathways played crucial functions in reproductive endocrinology (Fig. 5b). The coexpression network in the darkgreen module was further explored. After filtering out weak connections with weights less than 0.25, we generated a network consisting of 89 genes, including 3 hub genes, *CDC42* (Unigene0074964, *cell division control protein 42*), *SLC27A6* (Unigene0008858, *long-chain fatty acid transport protein 6*) and *ACBP* (Unigene0038759, *acyl-CoA binding protein*) (Fig. 5c and Additional file 9: Table S8). Among the three hub genes, *CDC42* connected with genes in multiple pathways, including *GnRHR2* (Unigene0054690), *PTGFR* (*prostaglandin F receptor*, Unigene0036737), and *RDH12* (Unigene0050327), suggested the potential cascade regulation of the seasonal reproduction in *G. przewalskii*. The network included interactions among known reproduction-related genes, such as the connection between *BMP7* (Unigene0017986) and *FSHR* (*FSH receptor*, Unigene0088176). Links among fish pheromone receptor genes, *PTGFR*, *PTGER4* (*prostaglandin E2 receptor EP4*, Unigene0061799) and *GnRHR2* were depicted, suggesting pheromone had potential effects on the seasonal reproduction in *G. przewalskii* (Fig. 5c and Additional file 8: Table S9). This gene network indicated sophisticated control of seasonal reproduction via transcriptional regulation.

## Discussion

Reproduction in most fishes is a seasonal phenomenon that occurs at a precise time of the year to ensure the survival of the offspring. The timing of the spawning migration is tightly controlled by the coordination of internal and external signals. Studies of the genetic and physiological bases of reproduction have been carried out in several fish species, and the results suggested both universality and diversity in the modulation of seasonal breeding [11, 22–26]. Among environmental cues, photoperiod is considered the primary and most reliable determinant of the timing of reproduction via neuroendocrine regulation [27, 28]. *G. przewalskii* inhabits in a region with evident seasonality. Long-term observations have demonstrated that the water temperature in this region shows significant fluctuation in the spring and early summer due to the sunshine, wind and precipitation. However, the day length peaks from May to Aug (Fig. 1c) [20, 21]. Therefore, we proposed that increased day length played the critical role in regulating seasonal reproduction in Tibetan highland fish. In the current study, we compared reproductively active and dormant *G. przewalskii* at the transcriptomic level, aiming to uncover the regulatory mechanisms that synchronize environmental cues and reproduction. The high quality of the reference transcriptome laid a foundation for the study of the molecular basis of seasonal breeding in *G. przewalskii*.

### Potential interplay between photoperiod and reproduction in *G. przewalskii*

Several neuroendocrine molecules were identified in RS brain samples, which was the possible outcome of the increased day-length in RS. DIO2, the key enzyme in the conversion of thyroid hormone to its bioactive form [8, 29], is induced by LD exposure in birds and mammals during the RS [30, 31]. The enhanced transcription of *DIO2* found in reproductively active fishes may result from the increased day-length, based on the similar regulatory mechanisms in other vertebrates. This result highlights its potential role in facilitating seasonal reproduction via photoperiodicity in *G. przewalskii*. Meanwhile, several neuroendocrine receptor genes that have been reported to control sexual maturation and reproduction in other species were stimulated in *G. przewalskii* during the RS. For example, massive studies have confirmed that Kiss1 and its receptor mediate the photoperiodic control and sexual maturation in male fishes and mice via promoting GnRH production [32, 33]. In reproductively active male *G. przewalskii*, the activation of *Kiss1r* during the RS may bridge the gap from LD sensing to gene expression in GnRH signaling. It has been reported that neuroendocrine receptor genes, *NPYR*, *nAChR9*, *RXFP2*, *GALR2* and *OXTR* exhibit robust circadian expression patterns and regulate HPG axis in many species [34–38]. The activation of these genes in female individuals suggests their gender-specific roles in regulation of genes in GnRH in *G. przewalskii* through the increased day-length in RS [34–38].

The function of GnRH in reproduction has been well established in a variety of species. GnRH promotes the production of LH and FSH, which in turn result in gonad development and breeding behaviors [9, 39, 40]. The simultaneous upregulation of *GnRH2* and *LHβ* in brain, as well as that of *testosterone 17-β-dehydrogenase 3* and *estradiol 17-β-dehydrogenase 1* in gonad, highlights the importance of the GnRH signaling pathway in the coordination of sexual hormone synthesis in *G. przewalskii* during the RS. Overall, the transcriptomic analysis indicated the involvement of photoperiodic and neuroendocrine regulation in the seasonal reproduction of *G. przewalskii*.

Melatonin is known to control the timing of reproduction by influencing GnRH production in many species, including fish [41]. However, we did not observe DEGs in melatonin synthesis in the current study, indicating that melatonin probably was not engaged in directing seasonal reproduction in *G. przewalskii*. In addition to melatonin, dopamine, GABA and NPY were thought to regulate gonadal development in fish [10], therefore, we considered that other neuroendocrine molecules, such as NPY, acetylcholine, relaxin, galanin and oxytocin, contributed to seasonal breeding in *G. przewalskii*.

### Dual functions of retinol metabolism in male *G. przewalskii* reproductive regulation

The functional enrichment analysis indicated that retinol metabolism probably exerted an important function in controlling reproduction in male *G. przewalskii*, due to the activation of the pathway in both brain and testis during RS. Retinol metabolism involves a series of reactions that convert dietary vitamin A to *all-trans* retinoic acid (RA), the cellular active metabolite [42]. First, vitamin A is processed to *all-trans* retinol, which is stored predominantly in the liver. In response to the body’s requirements, retinol is secreted from the liver and delivered to target tissues, where RA is generated [43]. RA binds to its receptors, retinol X receptor α (RXRA) and retinol X receptor γ (RXRG), which exert diverse functions depending on the tissue and cell types [39, 43–45]. Accumulated evidence suggests that RA and its receptors control spermatogenesis. RALDH is the rate-limiting enzyme for RA biosynthesis [46]. Mice deficient in genes encoding RALDH and RA receptors showed phenotypes of low RA levels in testis and impaired spermatogenesis, indicating that RA is crucial for male reproduction [47, 48]. In the current study, we observed that genes responsible for RA synthesis were stimulated in RS testis, probably causing elevated RA concentration. Given this information and the increased transcription of RA receptor genes, it was reasonable to speculate that the upregulation of genes in retinol metabolism and RA signaling may contribute to the seasonal formation of sperm in male *G. przewalskii*.

On the other hand, retinol metabolism regulated the seasonal reproduction through the deep brain photoreceptors in lower vertebrates, by influencing neurogenesis, locomotion, and synaptic plasticity [49]. It is well accepted that nonmammalian vertebrates detect light via deep brain photoreceptors lying outside the retina [50]. Vertebrate ancient (VA) opsin and *11-cis*-retinal form the photopigments that respond to photoperiodism in the hypothalamus of birds [51]. In fish species, all components of the photoperiodic transduction machinery are integrated into a brain region called SV, where opsin family genes are expressed [2]. The activation of genes in retinal synthesis suggests that they probably participate into the photoperiodic response by interacting with opsin in male *G. przewalskii* brain.

### Molecular network controlling seasonal reproduction in *G. przewalskii*

Co-expression network analysis uncovered interactions among the genes controlling seasonal reproduction in *G. przewalskii*. For example, a connection between *BMP7* and *FSHR* in *G. przewalskii* was revealed, in accordance with a previous finding showing a direct increase in *FSHR* expression by *BMP7* activation in human cell lines [52]. This result improved our confidence in the gene interactions in the network and indicated the universality in reproductive regulation between fish and mammals. In addition, the hub gene *CDC42* has been reported to modulate reproduction by stimulation of LH synthesis, oocyte meiotic maturation and fertilization in mammals [53, 54]. We also noticed the connections of *CDC42* with genes in GnRH signaling, retinol metabolism and oocyte maturation in the network, emphasizing its importance in coordinating the photoperiodicity, the HPG axis and gonad development in *G. przewalskii*. Moreover, it was worth noting the involvement of prostaglandin receptor genes *PTGER4* and *PTGFR* in the network, which interacted with *PGTR*, *GnRHR2* and *FSHR*. Prostaglandins are the major water-borne, hormonally derived sex pheromones in fishes. They induce reproductive behavior by interacting with receptors [23, 55]. Our results indicated they were co-regulated in the reproductively active male gonad, highlighting the potential feedback response in controlling the spawning migration in male population. Further works are required to untangle the role of pheromone in seasonal breeding of *G. przewalskii*.

## Conclusions

The current study was the first attempt to investigate the molecular regulation of seasonal reproduction in Tibetan highland fish. The activation of *DIO2* by the LD of the summer may influence sex hormone synthesis in *G. przewalskii*. The upregulation of neuroactive ligand-receptor genes was involved in the response to day-length change, and these receptors probably switched on genes in GnRH signaling and steroid hormone synthesis. Genes in retinol metabolism were specifically stimulated in reproductively active brain and testis to manage phototransduction and spermatogenesis in male *G. przewalskii*. The network analysis identified *CDC42* as a hub due to its interactions with genes in multiple pathways. Meanwhile, the interaction among prostaglandin receptor genes with *GnRHR2* and *FSHR* suggested the potential feedback regulation in courtship behaviors in male *G. przewalskii*. Moreover, the well-designed condition controlled experiments are required to untangle effects of environmental factors, such as water temperature and salinity on gene expression variations during the reproductive migration in *G. przewalskii*. In conclusion, our results profiled the transcriptomic alterations between reproductively dormant and active fish, suggesting that the LD condition in the summer was the principal signal to induce genes in neuroactive ligand-receptors and retinol metabolism in female and male brain. The synchronized upregulation of genes in GnRH signaling and sex hormone synthesis may promote gonad development and spawning migration in *G. przewalskii*.

## Methods

### Sample collection and RNA isolation

Twelve reproductively active male (body weight = 102.6 ± 30.46 g) and female (body weight = 244.5 ± 51.64 g) *G. przewalskii* were captured in June 2015 in one of their spawning grounds, the Quanji River, which is connected to Lake Qinghai. Reproductively dormant male (body weight = 208.1 ± 60.11 g) and female (body weight = 197.8 ± 43.46 g) naked carp were collected in Lake Qinghai in November 2015. The field study was authorized by the Qinghai Provincal Bureau of Fishery. All the fish were sexually mature and over 3 years old. The age of each fish was estimated based on its anal scales, dorsal fin spines and otolith, according to a previous publication [56]. Fish samples were euthanized with 200 mg/L MS222 (Sigma, USA), and tissues were harvested and transferred immediately to liquid nitrogen for RNA extraction. The animal experiment was conducted following the procedures and guidelines described in the “Guidelines for Animal Care and Use” manual approved by the Animal Care and Use Committee, Northwest Institute of Plateau Biology, Chinese Academy of Sciences.

Total RNA was purified from the brains and gonads of both male and female *G. przewalskii* in RS and NRS using TRIzol Reagent (Invitrogen, USA), followed by treatment with RNase free DNase I (Thermo Scientific, USA) to remove DNA contamination. The quality and quantity of RNA were verified with 1% agarose gel, NanoPhotometer® spectrophotometer (Implen, USA), Qubit® 2.0 Fluorometer (Life Technologies, USA), and Agilent Bioanalyzer 2100 system (Agilent Technologies, USA).

### Library preparation and sequencing

For each sample, we purified RNA from both brain and gonad tissues to construct 2 sequencing libraries. The sequencing included 3 biological replicates of two genders collected in reproductive and nonreproductive seasons. In total, 24 libraries were sequenced. A total of 1.5 μg RNA was used for sequencing library construction. Sequencing libraries were built using the NEBNext® Ultra™ RNA Library Prep Kit for Illumina® (NEB, USA) according to the manufacturer’s instructions. The sequencing tags were ligated to the sequence library of each sample. Briefly, mRNAs were purified from total RNAs using poly-T oligo-attached magnetic beads, which were fragmented using divalent cations in NEBNext First Strand Synthesis Reaction Buffer (5×). The first-strand cDNA was synthesized using random hexamer primer and M-MuLV Reverse Transcriptase (RNase H^−^), and the second-strand cDNA was synthesized using DNA polymerase I and RNase H^−^. Blunt ends were generated, and then, A was added at the 3′ ends, followed by the ligation of NEBNext adaptors for hybridization. The library molecules were selected for cDNA fragments of 150–200 bp. For library amplification, the PCR was carried out with Phusion High-Fidelity DNA polymerase (NEB, USA), universal PCR primers and index (X) primer. After purification of the PCR products, the library quality was measured by Qubit® 2.0 Fluorometer (Life Technologies, USA) as well as Agilent Bioanalyzer 2100 system (Agilent Technologies, USA). In total, 24 RNA-seq libraries were constructed and clustered using the cBot Cluster Generation System by TruSeq PE Cluster Kit v3-cBot-HS (Illumina, USA) based on the manufacturer’s recommendations. Finally, the 24 libraries were sequenced with an Illumina HiSeq™ 4000 platform by GeneDenovo Biotechnology Co. (Guangzhou, China).

### De novo assembly and annotation

The raw data were separated and processed to remove adapters, poly-N containing reads (N% > 10%) and low-quality reads (quality score ≤ 10) using custom Perl scripts. Trinity software was applied with the default parameters to assemble clean reads [57]. The assembled transcriptome was annotated by 4 public databases, including Nr, KOG/COG (http://www.ncbi.nlm.nih.gov/COG/), Swiss-Prot (http://www.expasy.ch/sprot) and KEGG (http://www.genome.jp/kegg/) [58]. We used the BLASTx program (http://www.ncbi.nlm.nih.gov/BLAST/) with an E-value less than 1e-5 as the threshold.

### DEG expression analysis

Gene expression levels were estimated using RSEM, and RPKM was calculated to represent the expression level for each gene [59]. To obtain the gene expression value for each sample, we first removed rRNA reads from the clean reads. The filtered, high-quality clean reads were aligned to the assembled reference transcriptome using Bowtie2 [60], and read counts for each gene were recorded. The EdgeR package (v3.20.1) was used to analyze the read counts to determine differential expression. To control false discovery rate (FDR), *p*-values were adjusted based on Benjamini and Hochberg’s approach. Finally, genes with an adjusted p-value less than 0.05 and an absolute value of fold change greater than 2 were defined as DEGs [61]. A clustering analysis was performed using pheatmap package in R, based on the RPKM of the DEGs.

### DEG enrichment analysis

Gene Ontology (GO) enrichment analysis for all DEGs was performed separately for down- and up-regulated genes by Blast2GO software [62]. The functional classification of unigenes was performed using WEGO software [63]. The significantly enriched GO terms were considered those with a p-value less than 0.05. The DEG-related KEGG pathways were obtained using KOBAS software [64], and pathways with a p-value less than 0.05 were defined as significantly enriched pathways.

### Gene expression validation by RT-qPCR

The cDNA was synthesized using 1 μg total RNA with the M-Mul First Strand cDNA Synthesis Kit (Sangon Biotech, China). In the reverse transcription (RT) step, PCR water was used to replace RNA samples as the RT control. The PCR mixture included diluted RT products (1:5), SGExcel FastSYBR Mixture (Sangon Biotech, China) with forward and reverse primers in a final volume of 20 μl, which was incubated in ABI ViiA™ 7 platform (Applied Biosystems, USA). For each gene, 3 fish samples of similar weights were selected within each group as biological replicates to perform RT-qPCR validation. The RT-qPCR experiment was repeated 3 times as 3 independent experiments. The RT-qPCR conditions were as follows: initial incubation at 50 °C for 2 min; 95 °C for 5 min; 40 cycles at 95 °C for 5 s and an annealing temperature for 30 s; 95 °C for 10 s; melt curve detection of 65 °C for 5 s to 95 °C with an increment of 0.5 °C. Two types of control were applied, the RT control and a blank of PCR water, and no amplicon was observed in the real-time PCR controls. The gene expression level for each sample was calculated using the ΔΔCt method using as 18S rRNA internal control to normalize the data [65]. We calculated the logFoldChange of 11 unigenes between the RS and NRS across FB, FG, MB and MG for both RNA-seq and RT-qPCR data. The Pearson correlation coefficient was estimated using all 44 pairs of data using the correlation function in the R environment. The primer information is listed in Additional file 10: Table S1.

### Weighted gene coexpression network analysis

To identify potential regulatory networks in the seasonal reproduction, we performed the WGCNA package (v1.47) in R [66–68]. The gene expression data was imported into WGCNA to generate coexpression modules using the blockwiseModule function for automatic network construction with default parameters. Modules were defined as clusters of highly interconnected genes. The eigengene of the module was the first principal component of a cluster of genes within the module, which represented the module’s gene expression profile. The grey module included genes that cannot be classified into any other modules. Genes in each module were subjected to Gene Ontolgy (GO) analysis and KEGG pathway enrichment tests. The intramodular connectivity of each gene was calculated using the softConnectivity function, and genes with high connectivity were considered as the hub genes. The networks were visualized using a free online platform (http://www.omicshare.com/tools).

## Additional files

Additional file 1: **Table S2.** KOG annotation of the *G. przewalskii* reference transcriptome. (XLSX 599 kb)

Additional file 2: **Table S3.** KEGG annotation of the *G. przewalskii* reference transcriptome. (XLSX 180 kb)

Additional file 3: **Table S4.** DEGs in female brain. (XLSX 384 kb)

Additional file 4: **Table S5.** DEGs in male brain. (XLSX 169 kb)

Additional file 5: **Table S6.** DEGs in female gonad. (XLSX 275 kb)

Additional file 6: **Table S7.** DEGs in male gonad. (XLSX 2574 kb)

Additional file 7: **Figure S1.** Eigengene expression pattern of each module. The a-axis represented the individual samples. The y-axis denoted the eigengene expression. The eigengene was the first principal component of a cluster of genes within the module, which represented the module’s gene expression profile. The grey module contained genes that cannot be grouped together in any other module, therefore, the eigengene expression was not calculated and plotted. (PDF 284 kb)

Additional file 8: **Table S9.** Correlation coefficients between modules and sample groups with corresponding *p*-values. (XLSX 51 kb)

Additional file 9: **Table S8.** Gene interactions in the darkgreen module. (XLSX 279 kb)

Additional file 10: **Table S1.** Primer information. (PDF 141 kb)

## Abbreviations

DEG: Differentially expressed gene
FDR: False discovery ratio
FSH: Follicle-stimulating hormone
GALR2: Galanin receptor 2
GnRH: Gonadotropin-releasing hormone
GO: Gene ontology
HPG axis: Hypothalamic-pituitary-gonadal axis
Kiss1r: KISS1 receptor
LD: Long-day
LH: Luteinizing hormone
nAChR9: Acetylcholine receptor 9
NPYR: Neuropeptide Y receptor
NRS: Nonreproductive season
OXTR: Oxytocin receptor
RA: Retinoic acid
RS: Reproductive season
RXFP2: Relaxin receptor 2
RXRA: Retinol X receptor α
RXRG: Retinol X receptor γ
TP: Tibetan plateau
WGCNA: Weighted gene coexpression network analysis
