# Supplementary figures and images for: Transcriptomic profiling reveals molecular regulation of seasonal reproduction in Tibetan highland fish, Gymnocypris przewalskii
Source: BMC Genomics. 2019 Jan 3;20:2. doi: 10.1186/s12864-018-5358-6 (PMC6318897; doi:10.1186/s12864-018-5358-6)

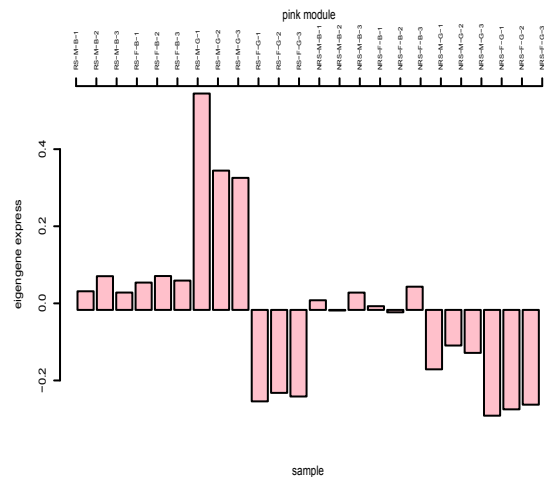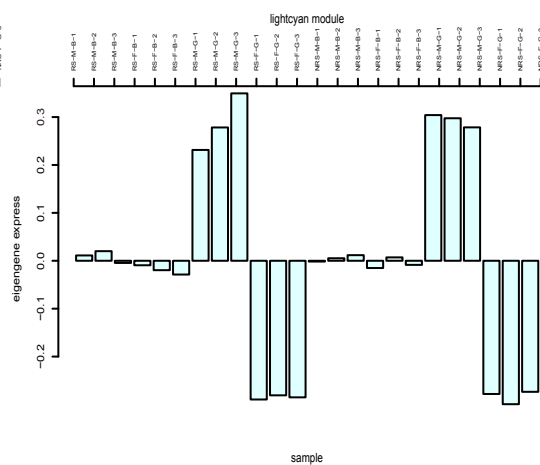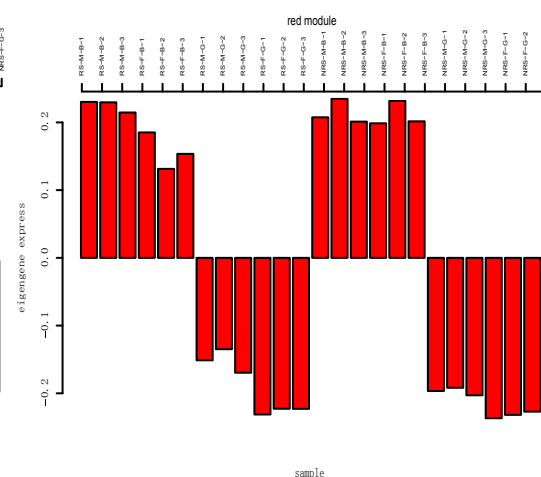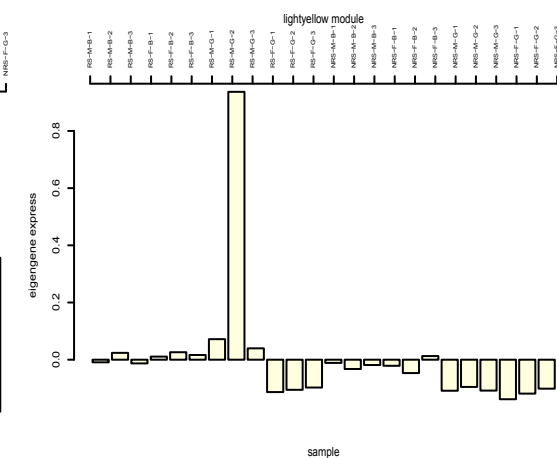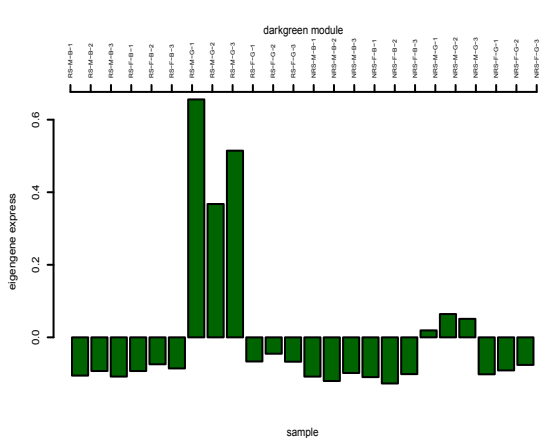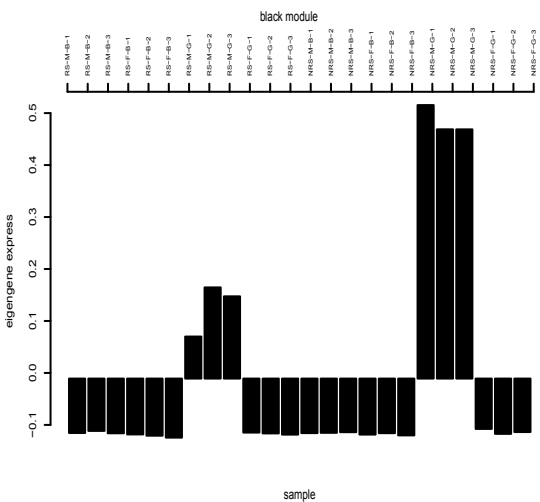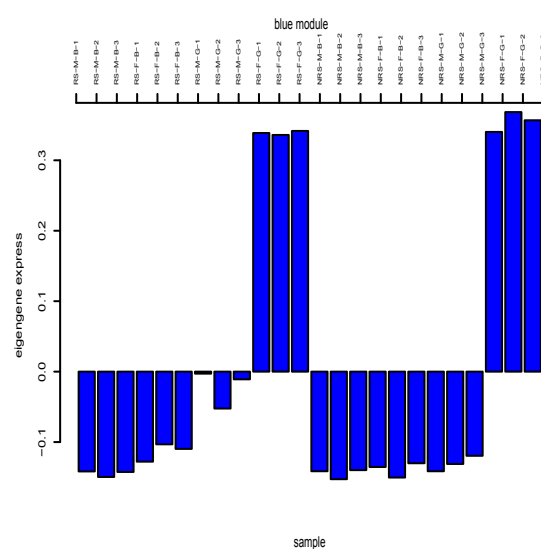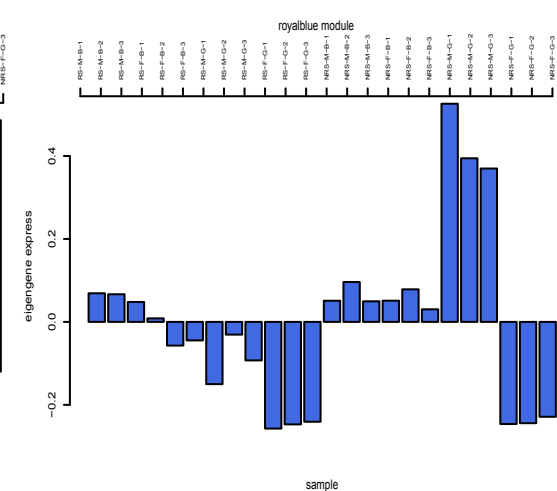

Supplement: Supplementary file 7 — Figure S1. Eigengene expression pattern of each module. The a-axis represented the individual samples. The y-axis denoted the eigengene expression. The eigengene was the first principal component of a cluster of genes within the module, which represented the module’s gene expression profile. The grey module contained genes that cannot be grouped together in any other module, therefore, the eigengene expression was not calculated and plotted. (PDF 284 kb) [file 12864_2018_5358_MOESM7_ESM.pdf]
