# Supplementary material for: Transcriptomic profiling reveals molecular regulation of seasonal reproduction in Tibetan highland fish, Gymnocypris przewalskii
Source: BMC Genomics. 2019 Jan 3;20:2. doi: 10.1186/s12864-018-5358-6 (PMC6318897; doi:10.1186/s12864-018-5358-6)
Supplement: Supplementary file 10 — Table S1. Primer information. (PDF 141 kb) [file 12864_2018_5358_MOESM10_ESM.pdf]

Table S1 Primer information

Symbol	GeneID	Annotation	Primer sequence (5'-3')
HSD17B3	Unigene0005059	testosterone 17-β-dehydrogenase 3	TTGCCAAAATACCCTGTCC
			ATGTCTGGCTTCTGATGTCCT
NPY	Unigene0045418	neuropeptide Y	AGGGTGGGACTCTGTTTCA
			AGGAGCTCGCCAAGTATTATT
GnRH2	Unigene0047142	GnRH2	TGATGGGGATGTTGCTGTGTCTAA
			GAGGGCATCCAGCAGTATTGTCTT
SST1A	Unigene0049479	somatostatin-1A	AGACACCAAACTCCGCCAACTT
			GACGCACTTCATCTTTCTCCACAG
CCK	Unigene0050835	cholecystokinin	GCTGCGGGGTTCTTCATCATC
			TCCACCAGCAGTTGCCTTTCTC
IGF2	Unigene0057572	insulin-like growth factor II	TGCGCTCCGATGTCCTCAAA
			TGGCAGTCCTCAACAACCCTTCT
Kiss1r	Unigene0009160	KISS1 receptor	CATGGAGCGGAGGCAGTTT
			GTGTCAGTAGTTGTCGCTTCATTT
LHR	Unigene0086623	luteinizing hormone receptor	ATGTGGATCCAGCAATGTC
			AGGGATGGATCTGTTCTCAC
GTHb2	Unigene0104459	gonadotropin subunit β-2	GTAAGTGCACACGTGTTGATAGAC
			AATGAGACTGTAGCTGTGGAAAAG
OXTR	Unigene0049178	oxytocin receptor	CAGTCGCGGATGTACTATTT
			GTCCATAAAAGCGAAAGGTG
HSD17B1	Unigene0063161	estradiol 17- β-dehydrogenase 1	CAGTCGCGGATGTACTATTT
			GTCCATAAAAGCGAAAGGTG
